# *ADAR* Expression and Single Nucleotide Variants in Multiple Sclerosis Patients Affect the Response to Interferon Beta Therapy

**DOI:** 10.1055/s-0043-1771001

**Published:** 2023-07-10

**Authors:** Fatemeh Fakhr, Vahid Shaygannejad, Mehdi Khorrami, Leila Saberi, Omid Mirmosayyeb, Erfan Sadeghi, Majid Kheirollahi

**Affiliations:** 1 Department of Genetics and Molecular Biology, Faculty of Medicine, Isfahan University of Medical Sciences, Isfahan, Iran; 2 Department of Neurosciences Research Center, Alzahra Research Institute, Isfahan University of Medical Sciences, Isfahan, Iran; 3 Department of Noncommunicable Diseases Research Center, Fasa University of Medical Sciences, Fasa, Iran; 4 Department of Biostatistics and Epidemiology, Faculty of Health, Isfahan University of Medical Sciences, Isfahan, Iran

**Keywords:** multiple sclerosis, interferon-β, ADAR, rs2229857

## Abstract

Interferon (IFN)-β is the first-line disease management choice in multiple sclerosis (MS) with profound effects; however, in up to 50% of patients, clinical response does not occur. Ascertaining the responding state, need a long-term clinical follow-up, and this may lead to delay in use of other effective medications. IFN-induced cascade and its regulation is considered to play a major role in MS. Adenosine deaminase, RNA-specific (ADAR) dysregulation is important to IFN signaling pathway as an activity suppressor. Hence, we investigated the expression of *ADAR* and its single nucleotide variants of rs2229857 association with response to IFN-β in relapsing-remitting MS patients. mRNA levels and genotyping of rs2229857 in 167 MS patients were investigated via SYBR Green real-time (RT)-quantitative polymerase chain reaction and high-resolution melting RT PCR, respectively. The allele-A in rs2229857 and higher expression of *ADAR* were associated with poor response to IFN-β. Two response groups were significantly different in terms of annualized relapse rate, first symptoms, first extended disability status scale (EDSS), current EDSS, and the MS severity score. According to this study's findings, assessment of transcript levels and also variants in ADAR may be useful in identifying patients' response to IFN-β before starting treatment. Further investigations are needed to determine the potency of ADAR to be a predictive biomarker in drug responsiveness.

## Introduction

Multiple sclerosis (MS) is known to be an inflammatory and autoimmune condition of the central nervous system that mostly affects young adults with a not fully known etiology. 1 MS is a multifactorial disease that originates from interactions between genes and the environment with a gender ratio from 2/1 to 3/1 (women/men) in many countries. 2 MS is clinically heterogeneous and can be classified into three main subgroups: relapsing-remitting (RR) that is the most prevalent type, secondary progressive, and primary progressive. Regardless of the advent of new disease-modifying therapies such as fingolimod and natalizumab, interferon (IFN)-β and glatiramer acetate (GA) remain the first-line disease management option with proven major impacts according to their safety. 3 However, clinical response does not occur in up to 50% of patients. 4 Assessing treatment response to IFN-β can take 1 to 2 years follow-up, and this can lead to the ineffective course of treatment, in addition to extra cost on the patients and significant delay in the use of other effective secondary medications. 5 MS is a chronic disease with heterogeneity in course and clinical presentation, making it difficult to prescribe the right medication. These observations highlight the importance of finding pharmacological biomarkers that could improve current knowledge in drug responsiveness. 6 So far, there is no reliable molecular marker that could accurately predict the treatment response. 7

Several genomic studies demonstrated several candidate genetic variants that can be used as simple and cost-effective predictive biomarkers in IFN-β response. It is well known that human leukocyte antigen (HLA) class II alleles on chromosome 6p21 play a major role in genetic predisposition to MS, mainly HLA-DR and DQ alleles (DRB1*1501, DRB5*0101, DQA1*0102, and DQB1*0602). 8 Some of studies validated the association of HLA DRB1*1501 with better response to GA but not IFN-β, and this impact is rather moderate. It is assumed that the effect of IFN-β is principally mediated by the type I IFN response pathway and not by major histocompatibility complex class II molecules that makes the lack of an impact of HLA DRB1*1501 on IFN-β response. 4 9 Several investigations approve the downregulation of IFN-β production in MS patients. Consequently, the production level of IFN-β-induced genes is decreased. 10 11 12 Although some studies defined a subgroup of patients who show increased activation of type I IFN signaling pathway and upregulated expression of genes that are stimulated by IFN (ISGs), 13 14 this subtype does not respond well to IFN-β therapy as a result of its intrinsic higher activity of downstream signaling which is refractory to exogenous IFN-β. 14 One of ISGs that is directly induced by activation of IFN signaling cascade is adenosine deaminase, RNA-specific (ADAR), which has an inhibitory role against adverse effects of IFN pathway upregulation. 15 16 17 Also, many researches have shown that ADAR is differentially expressed in autoimmune diseases such as MS. 18 19 20 Other experiments evaluating the importance of single nucleotide polymorphisms (SNPs) in ISGs and other genes involved in MS have been done in parallel with gene expression analyses. According to these genome-wide association studies (GWASs), single nucleotide variants in *ADAR* gene has strong association with IFN-β response. 21 22 23 The human *ADAR1* is located on chromosome 1 band q21.1–21.2 and its transcription regulated by multiple promoters, which one of them is induced by IFN, and the others are constitutively active. Transcripts are translated into two different-length ADAR1 proteins, an IFN-inducible (p150) and a constitutively expressed (p110) isoform. ADAR p150 is an A-to-I editor of cytoplasmic viral double-stranded RNAs (dsRNAs) that modifies them to become an active or inactivated virus. This process helps the innate immune system to distinguish between intracellular dsRNAs and dsRNAs from viruses. 15 ADAR1 is implicated in type I IFN response pathway as a negative regulator by suppressing further induction of ISGs. 16 The aim of this study is to assess the effect of changes in *ADAR* gene and mRNA levels on making differences between patients in response to treatment.

## Materials and Methods

### Patients

This study was performed on a total of 167 (146 females and 21 males) RRMS patients according to McDonald's criteria, before treatment and after characterizing as IFN-β responders ( *n* = 71) and nonresponders ( *n* = 96) in the MS department of Kashani Hospital, affiliated to Isfahan University of Medical Sciences. Our patients were classified into a low-risk group (score 0 or 1) and high-risk group (score 2 or 3) for a suboptimal response after 6 to 15 months follow-up of INF-β treatment. The study was approved by the ethical committee of the Isfahan University of Medical Sciences. All patients were on IFN-β therapy at least for 12 months and evaluated for extended disability status scale (EDSS), brain and spinal magnetic resonance imaging (MRI) findings and annualized relapse rate (ARR). The last relapse was measured 6 months after the EDSS assessment. Demographic and clinical information of patients recorded by face-to-face interviews. Patients were randomly selected to be of any age, gender, and from several ethnicities in the study, but we specified several exclusion criteria for them, such as: (1) history of neurological disease other than MS, (2) history of psychological disorders, (3) history of autoimmune and inflammatory disorders other than MS such as Crohn's disease, ulcerative colitis, (4) existence of chronic internal diseases affecting drug metabolism such as renal failure, liver failure, diabetes, thyroid disorders, and tuberculosis, and (5) history of smoking or alcohol consumption. These conditions were evaluated by two neurologists who had no prior knowledge of the genotypic profiles of the patients.

### DNA and RNA Extraction

A 5-mL whole blood was collected in EDTA tubes from patients before treatment to analyze gene expression, then 15 months after follow-up and defining responders and nonresponders to assess genotypes and expression data, when they had provided written informed consent. Genomic DNA was extracted by DNA extraction Kit (GeNet Bio, South Korea) and total RNA was isolated from blood using Hybrid-RTM blood RNA extraction Kit (GeneAll Biotechnology, South Korea) according to manufacturer's protocol. DNA and RNA concentrations were measured using Nanodrops WPA Biowave II Spectrophotometer (Bio chrome, United States), then DNA solutions were diluted to 10 ng/mL.

### High-Resolution Melting Real-Time Polymerase Chain Reaction

High-resolution melting real-time polymerase chain reaction (PCR) using Rotor-Gene Q instrument (QIAGENE) was performed using forward primer (5′ TGACAGACAAGAAGCGAGA 3′) and reverse primer (3′ ATGTGGGTATATTACAGGTG 5′) to amplify the DNA region containing the rs2229857 SNP (126bp) under the following condition: 95°C for 12 minutes followed by 40 cycles of 95°C for 1 second, 61°C for 20 seconds, and 72°C for 20 seconds. The temperature has been raised gradually from 65 to 95°C within 2 minutes. The software Rotor-Gene 6000 series version 1.7 was used to analyze the results. Chi-square test was employed for Hardy–Weinberg equilibrium and comparison of genotype and allele frequencies. Sanger sequencing was used to confirm the accuracy of the detected variant in at least 10%. Following primers designed via Primer 3 software (F: 5′- TGACAGACAAGAAGCGAGA -3′) and (R: 5′- ATGTGGGTATATTACAGGTG -3′) to amplify the region of interest, the PCR products (126bp) were subsequently visualized using 2% agarose gel and bidirectional sequencing was performed by on an ABI 3130 sequencer (Applied Biosystems). The sequences were compared with the ADAR1 gene reference sequence.

### Quantitative Real-Time PCR

cDNAs were synthesized by using the FIREScript RT cDNA Synthesis Kit (Solis BioDyne, Estonia). Allele ID 7 (Premier Biosoft, Palo Alto, United States) was used to design the specific primers for *ADAR* (F: 5′- CTGTGTCATTCCATCTGTATATCA-3′; R: 5′- TTGTGCCTTCTCCGTTCTC-3′) and *HPRT1* as reference gene (F: 5′- TATATCCAACACTTCG-3′; R: 5′- CTTTCCTTGGTCAGG-3′). Expression levels of *ADAR* and *HPRT1* mRNA were quantified by Corbett Rotor Gene 6000 machine (Corbett Life Science) using Applied Biosystems SYBR Green PCR Master Mix.

### Statistical Analysis

Data were presented by mean (standard deviation) or frequency (%) for quantitative and qualitative variables, respectively. Kolmogorov–Smirnov's test was conducted to assess for normality assumption. Independent *t* (Mann–Whitney) or chi-square (Fisher's exact) tests were used to compare variables between the study groups. To compare variables among genotypes, chi-square, analysis of variance (ANOVA), or Kruskal–Wallis' tests were used. Finally, crude and adjusted multinomial logistic regression models were performed to estimate the odds ratios of respond to IFN-β in genotypes. Correlation between variables was identified with Spearman's correlation analysis ( *p* ≤ 0.05 was considered as significant).

Minimum required sample size was determined by using G*Power software version 3.1.9.2 based on the allele frequencies of responder and nonresponder groups from a similar study (reference), considering α and β equal to 0.05 as types 1 and 2 errors (power = 95%). Exact—proportions: inequality, two independent groups (Fisher's exact test). Options: exact distribution. Analysis: a priori: computation of required sample size.

## Results

### Demographic and Clinical Information

Based on the univariate analyzes, two groups were significantly different in terms of genotypes, ARR, first symptoms, first EDSS, current EDSS, and MS severity score (MSSS). GA and AA genotypes, ocular, and other symptoms were more frequent in the nonresponder group. Moreover, number of individuals with ARR was, respectively, higher in nonresponder group. Furthermore, responder group had a lower mean level of first EDSS and current EDSS. The mean of MSSS also measured to provide a more precise criterion for evaluating the disease severity in response to treatment and observed that the MSSS was higher in nonresponders than responders ( Table 1 ).

**Table 1:** Demographic and clinical information based on the study group

		Responder	Nonresponder	*p* -Value
Sex	Male	9 (12.7%)	12 (12.5%)	0.973
	Female	62 (87.3%)	84 (87.5%)	
SNP	GG	45 (59.2%)	18 (17.8%)	< 0.001
	GA	23 (30.3%)	39 (38.6%)	
	AA	8 (10.5%)	44 (43.6%)	
Lesion load	1	20 (34.5%)	17 (21.0%)	0.207
	2	21 (36.2%)	35 (43.2%)	
	3	17 (29.3%)	29 (35.8%)	
Annualized relapse rate	No	52 (81.3%)	61 (67.0%)	0.049
	Yes	12 (18.8%)	30 (33.0%)	
First symptoms	Ocular	16 (25.8%)	27 (29.7%)	0.006
	Sensory movement	40 (64.5%)	38 (41.8%)	
	Other	6 (9.7%)	26 (28.6%)	
Atrophy	No	10 (16.4%)	25 (30.5%)	0.053
	Yes	51 (83.6%)	57 (69.5%)	
Age		33.58 (7.91)	35.51 (10.18)	0.185 a
Height		164.37 (7.02)	163.86 (7.59)	0.663 a
Weight		63.48 (11.79)	66.02 (12.99)	0.196 a
Body mass index		23.47 (3.95)	24.50 (3.87)	0.094 a
First EDSS		1.44 (0.95)	1.95 (1.14)	0.003 a
Current EDSS		0.57 (0.94)	1.30 (1.53)	< 0.001 a
Progression index		−0.18 (0.24)	−0.14 (0.24)	0.272 b
Multiple sclerosis severity score		0.09 (0.16)	0.19 (0.27)	0.006 b

Abbreviations: EDSS, expanded disability status scale; SNP, single nucleotide polymorphism.

Note: Values are presented by number (%) or mean ± standard deviation.

a Estimated from independent *t* -test.

b Estimated from Mann–Whitney's *U* test.

### Analysis of Genotypic Associations with Other Variables

Individuals with GA and AA genotypes had higher ratio of ARR and mean level of first EDSS, compared with those with GG genotype ( Table 2 ). In comparison to EDSS, no significant correlation was found between the type of genotype and MSSS values. Maybe this is because of the differences in the feature and method of measurement of these two diseases progression criterion. MSSS corrects EDSS for duration by comparing the disability of a patient with the distribution of scores in cases with similar duration of the disease. Other variables found not to have a significant association with neither of the response variables.

**Table 2:** Association of genotype with study variables

		GG	GA	AA	*p* -Value
Sex	Male	9 (15.0%)	10 (17.2%)	2 (4.1%)	0.096
	Female	51 (85.0%)	48 (82.8%)	47 (95.9%)	
Lesion load	1	19 (36.5%)	11 (22.9%)	7 (17.9%)	0.060
	2	19 (36.5%)	24 (50.0%)	13 (33.3%)	
	3	14 (26.9%)	13 (27.1%)	19 (48.7%)	
Annualized relapse rate	No	45 (80.4%)	42 (77.8%)	26 (57.8%)	0.024
	Yes	11 (19.6%)	12 (22.2%)	19 (42.2%)	
First symptoms	Ocular	17 (30.9%)	11 (20.0%)	15 (34.9%)	0.119
	Sensory movement	31 (56.4%)	31 (56.4%)	16 (37.2%)	
	Other	7 (12.7%)	13 (23.6%)	12 (27.9%)	
Atrophy	No	10 (18.9%)	11 (22.9%)	14 (33.3%)	0.253
	Yes	43 (81.1%)	37 (77.1%)	28 (66.7%)	
Age		33.70 (9.03)	33.98 (8.38)	36.73 (10.47)	0.185 a
Height		164.68 (7.69)	164.03 (7.33)	163.39 (6.97)	0.658 a
Weight		65.00 (12.37)	63.40 (13.04)	66.69 (12.08)	0.400 a
Body mass index		24.00 (4.53)	23.41 (3.36)	24.91 (3.68)	0.145 a
First EDSS		1.52 (1.16)	2.01 (0.95)	1.66 (1.12)	0.043 a
Current EDSS		0.82 (1.39)	1.16 (1.45)	1.00 (1.19)	0.398 a
Progression index		−0.17 (0.24)	−0.20 (0.29)	−0.11 (0.17)	0.162 b
MSSS		0.11 (0.18)	0.19 (0.27)	0.15 (0.23)	0.202 b

Abbreviations: EDSS, expanded disability status scale; MSSS, multiple sclerosis severity score.

a One-way analysis of variance.

b Kruskal–Wallis' test.

### Results of Genotypic and Allelic Distribution Analysis between Responders and Nonresponders

**Fig. 1:**
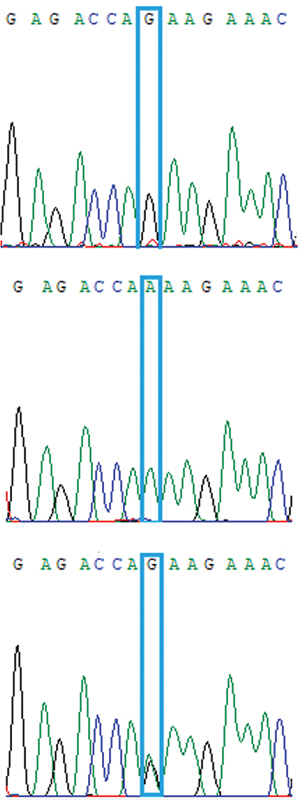
Sanger sequencing of samples for confirmation of HRM PCR. Sanger sequencing was used in 10% of samples for confirmation of HRM PCR. The three relevant genotypes of rs2229857 are GG, AA, and GA, respectively, from up to down. HRM, high-resolution melting; PCR, polymerase chain reaction.

Individuals with GA or AA genotypes have significantly lower odds to respond to IFN-β, compared with those with GG genotype ( Table 3 ). In this table, association of responding to IFN-β with genotypes was assessed using multinomial logistic regression model. Therefore, GG genotype was considered as the reference category, and GA and AA were compared with that. Chi-square test showed that allelic distribution between two groups of response were significantly different ( *p* -value < 0.001). In the nonresponders, the number of allele A was higher than the responders, and the number of allele G was higher in responders ( Table 4 ). The comparison of expected allelic and genotypic frequencies with observed frequencies showed that our study population is in Hardy–Weinberg equilibrium for rs2229857 ( *p* > 0.05). Ten percent of the samples were sent for Sanger sequencing to confirm the genotyping ( Fig. 1 ).

**Table 3:** Odds ratio and 95% confidence interval of responding to IFN-β in GA and AA genotypes, compared with GG

	GA	AA
Crude	0.236 (0.111–0.500)	0.073 (0.029–0.184)
Adjusted	0.208 (0.075–0.572)	0.038 (0.008–0.177)

Abbreviation: IFN, interferon.

Note: Adjusted for age, sex, body mass index, annualized relapse rate, first symptoms, atrophy, lesion load, and progression index.

**Table 4:** Chi-square test for comparison of allelic distribution between two groups

Comparison	Allele	Nonresponder	Responder	*p* -Value
Allele	A	127 (62.9%)	39 (25.6%)	<0.001
	G	75 (37.1%)	113 (74.4%)	

### Differential Expression Analysis of *ADAR* between Responders and Nonresponders

**Fig. 2:**
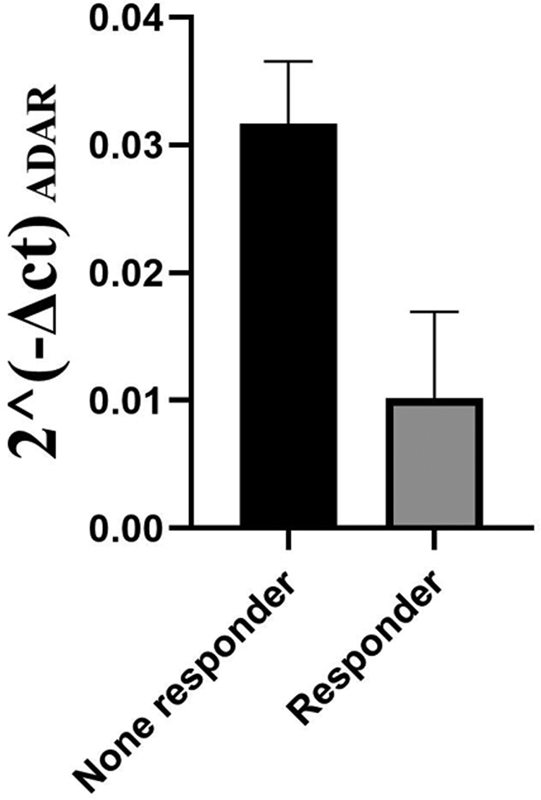
Relative ADAR expression rates between two groups of drug response. Quantitative real-time PCR was performed to measure expression rates and 2^-delta CT was used to show fold change analysis on the mean values. Responders and nonresponders were compared and 2^-delta CT was higher in nonresponders. Hence, ADAR had more expression in nonresponder group compared with responders. ADAR, Adenosine deaminase, RNA-specific; PCR, polymerase chain reaction.

**Fig. 3:**
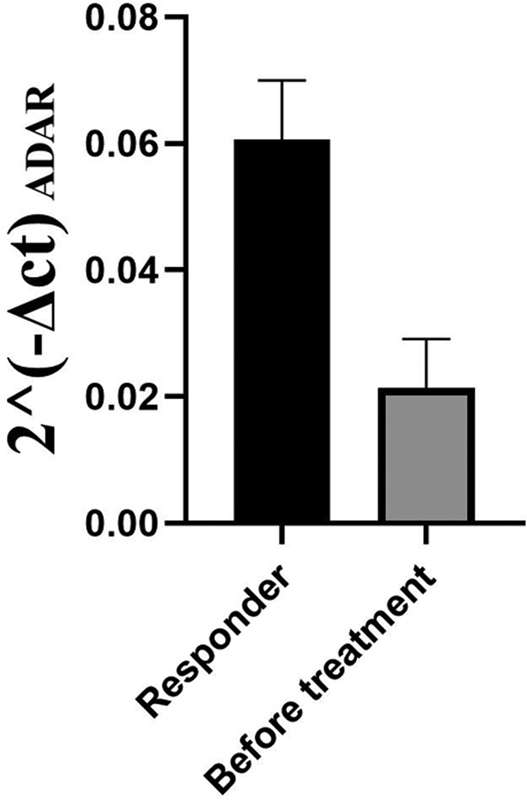
Comparison of ADAR expression in responders after 1 year follow-up with before starting drug therapy. Assessment of 2^-delta CT in responders comparing expression levels before beginning treatment and after a year follow-up showed elevated rates in ADAR expression in response to drug therapy. ADAR, Adenosine deaminase, RNA-specific.

According to Table 5 , results of RT-qPCR data from patients when comparing before treatment and after ∼1 year follow-up showed that there is a significant difference in *ADAR* mRNA expression levels between responders and nonresponders ( *p* = 0.001). Nonresponders had higher expression rates than responders (fold change = 2.1435) ( Fig. 2 ). Responders showed increased level of mRNAs after treatment follow-up compared with before starting therapy ( *p* < 0.001) ( Fig. 3 ). Although nonresponders did not show a significant change in expression rates after almost 1 year treatment ( *p* = 0.410), there was no considerable difference in expression levels between two age groups ( *p* = 0.563) and also males and females ( *p* = 0.632).

**Table 5:** ADAR expression assay between groups of patients

		Fold change	*p* -Value	Means of ΔCT ± SD
Age	<30	–	0.563	6.1 ± 0.7
	>30			5.7 ± 1.3
Gender	Male	–	0.632	5.1 ± 0.8
	Female			5.7 ± 1.1
Responder vs, nonresponder		2.1435	0.001	5.7 ± 1.3
				6.8 ± 0.6
Responder	After 1 y vs. before treatment	2.9563	<0.001	5.3 ± 1.2
				6.5 ± 0.9
Nonresponder	After 1 y vs. before treatment	0.8122	0.410	6.1 ± 0.8
				5.8 ± 1.1

Abbreviations: ADAR, adenosine deaminase, RNA-specific; SD, standard deviation.

### Power and Sample Size Calculation

Minimum required sample size calculating resulted in a total of 142 participants (71 in each group). Results of the power assessment are shown in Table 6 .

**Table 6:** Calculation of power of the study

**Input**	Tail(s)	Two
	Proportion p1	0.6920000
	Proportion p2	0.382
	α err prob	0.05
	Power (1 − β err prob)	0.95
	Allocation ratio N2/N1	1
**Output**	Sample size group 1	71
	Sample size group 2	71
	Total sample size	142
	Actual power	0.9525327
	Actual α	0.0319333

## Discussion

In many patients treated with IFN, the level of neutralizing antibodies increases, which negatively affects the response of these patients to drug therapy. But the impact of antibodies on the biological response to IFN-β may be detectable after 9 to 12 months, the clinical effects of neutralizing antibodies are not seen until ∼12 months after starting IFN-β therapy and the production of antibodies depends on the type of IFN-β consumed; IFN-β-1a is considered less immunogenic than others. Also, during prolonged IFN-β therapy, tolerance seems to occur over the long-term treatment and neutralizing antibody (NAb)-positive patients are likely to return to NAB-negative status. 24 For these reasons, antibody monitoring may be difficult and inconclusive to detect poor responsiveness to therapy. Although there is not a clear predictor of response to IFN-β therapy, relapses, disability progression, and MRI activity are the widely used methods for evaluating therapy response in many studies. 25

Several GWASs and expression data analyses were performed to find a reliable biomarker that indicates a strong correlation between the response to IFN-β therapy and the genomic alterations, but any validated biomarker, which could exactly predict an individual's response to current MS drugs, has not been reported yet. 4 26 Recent studies had failed to reach consensus on their findings. Inconsistencies between results reveal variation in description of responders and nonresponders, and these studies include populations with different ethnicities. They also use diverse methods. 4 Such findings need to be confirmed and repeated in different samples, even in other populations. 27 According to studies conducted on expression analysis, nonresponders are considered as a subtype of patients indicating higher levels of IFN-β signaling pathway activity, but responders show downregulated activity of the pathway. As a consequence, in nonresponder group, higher level of IFN-stimulated genes' transcription is expected, but in responder group, the opposite is true. 10 11 12 13 14 Several investigations claimed that *ADAR* that is one of important ISGs with regulatory role in IFN-β-induced downstream cascade is differentially expressed in autoimmune diseases such as MS 18 19 20 ; therefore, we decided to assess ADAR changes in relation to drug responsiveness in MS patients. We examined the alterations in ADAR mRNA production rates and association of rs2229857 with IFN-β response in MS patients in Isfahan, central part of Iran. We observed the significant differences in expression levels and allelic frequencies between responders and nonresponders. rs2229857 is a missense variant in coding region of *ADAR* gene and changes the sequence of amino acids (NM_001111.5(ADAR): c.1151A > G (p. Lys384Arg); this variant seems to play a modifying role in ADAR1 protein activity, although functional analyses are needed to make clear how it works. Our results, in line with Comabella et al's study 22 demonstrated that ADAR rs2229857 variants are significantly different between responders and nonresponders, and allele A is the risk allele for IFN-β response. Odds ratios (confidence interval = 95%) of AA and GA genotypes compared with GG (0.038, 0.208, respectively) were lower in respond to IFN-β. In the other hand, patients with AA and GA genotypes are more likely to be unresponsive. The ARR is the primary clinical indicator when assessing RRMS treatments in clinical trials since this result acts as a direct measure of the therapeutic benefits associated with RRMS patients and reflects a key aspect of the clinical burden of the disease. 28 29 We compared three genotypic groups in terms of ARR measurement criterion and found that the number of patients with increased relapse rates in 1 year was higher for the AA genotype, and the number of patients with GG genotype who did not have an increase in ARR was higher than other groups. These findings indicate that the AA genotype is associated with increased relapse rates during 1 year of IFN-β treatment. Comparing expression data of patients before starting treatment and after 15 months follow-up showed significant difference between responder and nonresponder groups. Nonresponders were twofold higher in mRNA levels than responders, but they did not exhibit considerable changes in expression rates before and 1 year after treatment follow-up. Maybe it is because of saturated IFN-β-induced cascade activity which is not responsive to exogenous IFN-β. 13 14 Responders represented increased levels of expression after ∼1 year treatment in comparison with before starting.

## Conclusion

ADAR, considering its expression and SNP variants, represents distinctive behavior in IFN-β responders compared with nonresponders in RRMS patients. Therefore, survey its alterations in patients' blood, before prescribing a method of therapy may be useful to choose a right decision. However, more experimental studies with a large cohort of patients and more accurate methods for additional validation are required to suggest ADAR as a prognostic marker for assessment of response to IFN-β therapy.
